# Burnout and Life Satisfaction among Healthcare Workers Related to the COVID-19 Pandemic (Silesia, Poland)

**DOI:** 10.1155/2024/9945392

**Published:** 2024-05-02

**Authors:** Daria Łaskawiec-Żuławińska, Mateusz Grajek, Karolina Krupa-Kotara, Patryk Szlacheta, Hasan Karacan, Mateusz Roszak, Beata Łabuz-Roszak, Ilona Korzonek-Szlacheta

**Affiliations:** ^1^Department of Cardiovascular Disease Prevention, Faculty of Public Health, Medical University of Silesia in Katowice, Piekarska 18, 41-902 Bytom, Poland; ^2^Department of Public Health, Faculty of Public Health in Bytom, Medical University of Silesia in Katowice, Piekarska 18, 41-902 Bytom, Poland; ^3^Department of Epidemiology, Department of Epidemiology and Biostatistics, Faculty of Public Health in Bytom, Medical University of Silesia in Katowice, Piekarska 18, 41-902 Bytom, Poland; ^4^Department of Basic Medical Sciences, Faculty of Public Health in Bytom, Medical University of Silesia in Katowice, Piekarska 18, 41-902 Bytom, Poland; ^5^Department of Eastern Languages and Literature, Cyprus Science University, Casaphani, Cyprus; ^6^Student Scientific Society at the Department of Neurology, Institute of Medical Sciences, University of Opole, Opole, Poland; ^7^Department of Neurology, Institute of Medical Sciences, University of Opole, 45-040 Opole, Poland

## Abstract

**Background:**

The phenomenon of burnout among healthcare workers during the COVID-19 pandemic is a widespread problem with several negative consequences for the healthcare system. The many stressors of the pandemic have led to an increased development of anxiety and depressive disorders in many healthcare workers. In addition, some manifested symptoms of the so-called postpandemic stress syndrome and the emergence of occupational burnout syndrome, commonly referred to as “COVID-19 burnout.” The aim of this study was to assess the burnout and life satisfaction of healthcare workers during the COVID-19 pandemic.

**Materials and Methods:**

The study was conducted in 2020-2022 among medical staff working in hospitals in Silesia, Poland. The instruments used to assess life satisfaction and burnout were the Satisfaction with Life Scale (SWLS) and the Maslach Burnout Inventory (MBI), which assesses three dimensions: emotional exhaustion (EE), depersonalisation (DEP), and sense of reduced professional accomplishment (SRPA).

**Results:**

The study group included 900 participants. There were 300 physicians (mean age 38 ± 7 years), 300 nurses (mean age 35 ± 6 years), and 300 paramedics (mean age 31 ± 5 years). Life satisfaction as measured by the SWLS was lowest among nurses and paramedics in 2021 and among doctors in 2022. Male respondents and those with fewer years of work had higher levels of life satisfaction. People with more years of work had higher scores in EE and DEP and lower scores in SRPA (*p* = 0.001). We found a negative correlation between life satisfaction and EE (*p* = 0.001), DEP (*p* = 0.001), and SRPA (*p* = 0.002).

**Conclusions:**

The results highlight the need for further research into the causes of burnout among medical professionals and the need for effective interventions to promote well-being and prevent burnout in this group.

## 1. Background

Modern healthcare is constantly facing new challenges and public expectations. Recently, one of the most significant challenges, with a huge impact on the mental health of a very important professional group such as healthcare workers, has been the spread of COVID-19 (coronavirus disease). Mental disorders among healthcare workers, particularly those on the front lines of the severe acute respiratory syndrome coronavirus 2 (SARS-CoV-2) pandemic, have become a global public health problem that threatens not only the mental health of individuals but also the safety of the general public. As such, it challenges the entire healthcare system in terms of the ability of healthcare workers suffering from poor mental health and the increasingly common phenomenon of professional burnout to provide quality care to patients.

Healthcare is a profession that takes a heavy toll on physical and mental health. Exposure of healthcare workers to occupational burnout has always been a common problem with a very wide range of effects [1, 2]. It is undoubtedly influenced by the specific nature of the work, including the constant presence of stressors associated with the enormous dynamics of the work, such as the need to make difficult decisions in a short period of time while other people's lives are at stake, or the constant confrontation with human suffering and death of patients. Shift work, i.e., irregular working hours and night work, which disrupts the circadian rhythm, also has an unfavourable effect [3].

The development of the COVID-19 pandemic has led to a change in working patterns in the healthcare sector, putting even more strain on the mental health of its workers. The cumulative effect of pandemic stressors has led to increased development of anxiety and depressive disorders in many healthcare workers. In addition, some manifested symptoms of the so-called postpandemic stress syndrome [4] and the emergence of occupational burnout syndrome, commonly referred to as “COVID-19 burnout” during the pandemic [5].

Postpandemic stress disorder is not currently recognised as a mental disorder, but it is a response to the traumatic event that the COVID-19 pandemic proved to be for many people. Its creator is psychotherapist O'Kane, who in 2021 defined it as a specific variant of PTSD (post-traumatic stress disorder). Its symptoms can include severe fear and anxiety, sleep disturbances, recurrent intrusive thoughts, and the depressive disorders mentioned above [6].

With regard to professional burnout, it should be mentioned that its issues have been addressed in the psychological literature for almost fifty years. The term was first used in 1974 by the American psychologist Freudenberger, who, while working in a centre for young drug addicts, noticed a progressive decline in motivation, energy, and commitment to their duties among volunteers who did charitable work for the centre. Translated, he defined burnout as follows: “a state of mental and physical exhaustion caused by working life” [7]. Over time, many definitions of burnout have emerged around the world. However, one of the most popular and widely used definitions was proposed by the American social psychologist Maslach, who, together with her colleagues, interviewed people in stressful professions, especially social service workers whose work is based on close interaction with others, i.e., psychiatric nurses, psychiatrists, and clinicians, among others. Maslach's main interest was the emotional stress experienced in the work environment and the assessment of coping [8]. She also created the Maslach Burnout Inventory (MBI) in 1981, which is still used today for the psychological assessment of job burnout. The questionnaire contains three subscales to measure relevant dimensions of burnout, which also correspond to the three components of the occupational burnout syndrome proposed by Maslach [9, 10]. These are as follows: emotional exhaustion, understood as an extreme lack of energy for life, chronic fatigue, and associated mood swings, manifested in the form of both mental and physical exhaustion; depersonalisation, associated with a sense of impersonality and an indifferent attitude to reality, manifested in a negative distance from others; and a reduction in the appraisal of personal accomplishment, understood as a sense of reduced professional effectiveness, manifested in a negative appraisal of one's work and professional competence [8, 10, 11].

During the COVID-19 pandemic, the phenomenon of occupational burnout was exacerbated by new stressors emerging in the work environment, which consequently exacerbated the complications for the Polish healthcare system [12]. Factors predisposing to anxiety symptoms in healthcare workers included increased exposure to COVID-19 compared to nonmedical workers, the risk of transmitting the infection to their loved ones, severe understaffing of medical personnel, and the associated pressure of extended on-call duty (often beyond one's strength) [12, 13]. Mental health and the risk of burnout among medical staff were also adversely affected by the admission of too many patients in relation to the number of medical staff, which only revealed the shortage of staff, and by the increased risk of death as a result of a not insignificant number of deaths, especially among patients with severe COVID-19 [14]. In addition, the epidemiological requirements for isolation and disinfection required healthcare workers to wear multiple layers of barrier isolation aprons, overalls, visors, and masks, which significantly increased their physical exertion and thus energy expenditure, often leading to oxygen deprivation. It is worth noting that in many medical facilities, the lack of the aforementioned personal protective equipment was also a common problem, preventing healthcare workers from being properly protected when working with patients with COVID-19. All of these factors placed a heavy burden on healthcare workers and took a toll not only on their mental health but also on their physical health [15].

A major problem associated with occupational burnout among healthcare workers is also the fact that the problem is not limited to the affected individual but also has an immeasurably negative impact on the functioning and effectiveness of the entire healthcare system, with dramatic consequences for the health security of society [16]. Research on such an important psychosocial phenomenon as occupational burnout is of paramount importance, as it draws attention to the widespread problem of declining effectiveness in one of the most important professional groups.

The psychophysical burden of healthcare workers and the associated risk of occupational burnout, due to the negative impact on professional competence and quality of work, should be constantly minimised. It is therefore undeniable that the mental health and well-being of healthcare workers should be taken into account in order to provide adequate healthcare to patients. This is very important because only physicians with so-called mental well-being can provide proper and professional medical care to patients, while maintaining the qualities of empathy and understanding and at the same time derive satisfaction from their work [17]. All of this makes the topic addressed highly topical and necessary, as the results of such studies can provide valuable guidance for clinicians and mental health managers.

Therefore, the aim of this study was to assess the burnout and life satisfaction of medical staff during the COVID-19 pandemic.

## 2. Material and Methods

The study was conducted in 2020-2022 among medical staff working in hospitals in Silesia, Poland. Inclusion criteria were as follows: at least one year of work experience in the current job and signed informed consent. The following instruments were used to assess life satisfaction and burnout: the Satisfaction with Life Scale (SWLS) and the Maslach Burnout Inventory (MBI). The following variables were also collected through the questionnaire: gender, age, occupation, and years of work.

The Satisfaction with Life Scale (SWLS) [18] is a short scale consisting of five statements designed to assess an individual's overall satisfaction with life as a whole. The SWLS does not focus on specific areas of life (such as health or finances), but rather on overall satisfaction. Respondents rate each statement on a 7-point Likert scale, ranging from 1 (strongly disagree) to 7 (strongly agree). Scores can range from 5 to 35 points. Higher scores indicate greater life satisfaction. The general interpretation is as follows: 5-9 points: dissatisfied with life; 10-14 points: below average satisfaction with life; 15-19 points: average satisfaction with life; 20-24 points: above-average satisfaction with life; 25-29 points: high satisfaction with life; and 30-35 points: very high satisfaction with life.

The Maslach Burnout Inventory-General Survey (MBI-GS) [19] is one of the most widely recognised tools for assessing occupational burnout. The scale focuses on three dimensions of burnout: emotional exhaustion (EE)—5 items, depersonalisation (DEP)—5 items, and a sense of reduced professional accomplishment (SRPA)—6 items. It is assumed that the MBI scale for each dimension takes values from 0 to 6 (with a higher score in EE and DEP and a lower score in SRPA indicating a higher risk of burnout). The results were reported as total values for each subscale and values converted to average scores (total score was divided by the number of items in the subscale). The maximum number of points to be obtained in each subscale was as follows: EE—30 points; DEP—30 points; and SRPA—36 points.

During the research period, the number of medical staff working in Silesian hospitals was approximately 60,000. According to the estimated expected sample size, with the established confidence level of 95% with an acceptable error of 5%, the minimum size of the study sample was set at 382 [20].

Computer-assisted web interviewing (CAWI) was used to collect data through a questionnaire created using the Google Forms programme. The survey was made available on the most popular social network in Poland (Fb) and distributed throughout Silesia. Participation in the study was voluntary. At the beginning, participants received detailed information about the study and its inclusion criteria. If they fulfilled them and decided to take part in the study, they declared their profession (doctor, nurse, and paramedic). Informed consent was required to proceed to the questionnaire. Participants completed the questionnaire on their own, without any assistance, at a place and time most convenient for them. The study was closed after receiving 900 complete questionnaires (300 in the physician group, 300 in the nurse group, and 300 in the paramedic group).

The study using the methodology described above was approved by the Bioethics Committee of the Silesian Medical University in Katowice (PCN/CBN/0052/KB/127/22).

Data were processed using Microsoft Excel 2019 and STATISTICA 13 (Stat Soft Poland). Results were considered statistically significant when *p* values were < 0.05. Measurable data were characterised by means (*X*s) and standard deviations (SDs). The significance of differences was tested using the Student's *t*-test for two groups and the ANOVA test for three or more groups. An analysis of the association between life satisfaction and job burnout was carried out using the Spearman coefficient.

## 3. Results

The study group consisted of 900 people. There were 300 physicians (mean age 38 ± 7 years), 300 nurses (mean age 35 ± 6 years), and 300 paramedics (mean age 31 ± 5 years). Among the doctors, both sexes were equally represented, with 50% women (*n* = 150) and 50% men (*n* = 150). Among the nurses, women dominated with 80% (*n* = 240). Among the paramedics, however, men were in the majority, accounting for 60% (*n* = 180).

Based on an analysis of life satisfaction using the SWLS, doctors and paramedics reported above-average life satisfaction in 2020 (23 ± 1.5 and 22 ± 1.6 points, respectively), whereas nurses reported average life satisfaction (19 ± 1.7 points). However, a decline was observed in the following years. The worst year for nurses and paramedics was 2021 and for doctors 2022. The results of the life satisfaction scale are shown in Table 1.

We found that the number of work years correlated negatively with life satisfaction in all the professions examined considering three years (2020, 2021, and 2022) (*p* = 0.001).

Regarding gender, men seem to be slightly more satisfied with their lives than women in all professions. The mean score for male physicians was 22 ± 1.4 points (compared to 21 ± 1.5 for women, *p* < 0.05), for male nurses −19 ± 1.9 (compared to 18 ± 1.8 for female nurses, *p* < 0.05), and for male paramedics −21 ± 1.6 (compared to 20 ± 1.7 for female paramedics, *p* < 0.05).

The results of the Maslach Burnout Inventory (MBI) scale are shown in Table 2. A comparison over three years showed that the mean scores for the EE and DEP scales were highest in 2021. The scores for nurses were the worst (indicating the highest risk of burnout) in each year in comparison to doctors and paramedics (*p* < 0.05).

Analysis considering all examined years revealed that respondents with more years of work had higher scores in EE and DEP and lower scores in SRPA (*p* = 0.001). When analysed by gender, women had higher scores in the EE category and lower scores in the DEP and SRPA categories compared to men (*p* = 0.001).

Finally, the association between life satisfaction and job burnout was analysed for all the professions and all the years. There was a strong negative correlation between life satisfaction and emotional exhaustion (*R* = −0.62, *p* = 0.001), a moderate negative correlation between life satisfaction and depersonalisation (*R* = −0.55, *p* = 0.001), and a moderate negative correlation between life satisfaction and a sense of reduced occupational accomplishment (*R* = −0.48, *p* = 0.002). Analyses indicated that the higher the level of professional burnout (in all three dimensions), the lower the level of life satisfaction among medical professionals.

## 4. Discussion

The results of our study confirm the impact of the COVID-19 pandemic on job burnout among medical professionals (doctors, nurses, and paramedics) and its association with life satisfaction.

An analysis of the SWLS scores and the emotional exhaustion dimension of the MBI showed a strong negative correlation. This means that higher levels of emotional exhaustion are associated with lower levels of life satisfaction among health professionals. Such a correlation underlines the serious psychological consequences associated with occupational exhaustion in this professional group. It was found that the higher the level of depersonalisation among health professionals, the lower their life satisfaction. Depersonalisation, which is one of the main indicators of professional burnout, can lead to feelings of dehumanisation, which have a negative impact on overall well-being. A moderate negative correlation between feelings of reduced job fulfilment and life satisfaction suggests that employees who feel less fulfilled in their job role are generally less satisfied with their lives. This may indicate the importance of job satisfaction for the overall well-being of health professionals.

The phenomenon of professional burnout is a widespread problem that has several negative consequences, not only for the individuals struggling with psychological problems but also for the healthcare system as a whole. Similar to our study, other researchers have analysed the impact of the COVID-19 pandemic on burnout among healthcare workers in different countries [21–33].

Morgantini et al. conducted a survey on job burnout among healthcare workers from 60 countries [21]. They found that more than half of the respondents reported emotional exhaustion and work-related burnout during the COVID-19 pandemic. The authors also analysed factors that increased the likelihood of healthcare workers developing burnout (heavy workload, exposure to COVID-19 patients, inadequate access to personal protective equipment, and fear of transmitting the infection to household members).

Similar to our study, the MBI questionnaire was used by Barello et al. among Italian healthcare workers (mainly nurses, but also physicians and other professionals) during the peak of the COVID-19 pandemic [22]. High emotional exhaustion was reported by 37% of Italian medics and depersonalisation by almost a quarter. The third component of burnout—a sense of reduced professional accomplishment—was experienced by about 15%. Total scores for the EE subscale in our study ranged from 20.0 ± 5.5 for paramedics in 2020 to 25.5 ± 4.5 for nurses in 2021 and were comparable to those obtained by Barello et al. (22.7 ± 12.1 points). On the other hand, our research's total scores for the DEP subscale ranged from 16.5 ± 5.0 for paramedics in 2020 to 21.5 ± 6.0 for nurses in 2021 and were much higher than those achieved by Italian authors (6.1 ± 5.7 points) indicating a higher risk of burnout in the examined group of Polish healthcare workers. The situation was similar for the SRPA subscale; our results indicated a higher risk of burnout (total score from 13.8 ± 4.8 for paramedics in 2022 to 19.2 ± 6.6 for nurses in 2021) than those described in paper by Barello et al. (37.5 ± 7.6 points; one should remember that higher total scores in EE and DEP subscales and a lower total score in SRPA subscale indicate a higher risk of burnout). Almost half of the Italian respondents experienced at least one of the symptoms, such as increased irritability, changes in eating habits, sleep problems, or even nervous breakdowns. Compared to other studies in Italy conducted before the pandemic outbreak, the problem of job burnout was much greater during COVID-19 pandemic [23, 24].

The prevalence of burnout during the COVID-19 pandemic and in the prepandemic period has been described by Butera et al. [27]. Similar to our study, the research involved nurses, but only those who had direct contact with COVID-19 patients (in hospital emergency departments (EDs) and intensive care units (ICUs)). The study showed that the pandemic had a greater impact on nurses working in ICUs, with up to almost 90% reporting a significant increase in their psychological workload following the pandemic outbreak, which significantly increased their risk of burnout. Similar situation was observed in our study; the risk of burnout in nurses was higher than in doctors and other professionals; and it was stable for three examined years. In addition, it is worth noting that in the paper by Butera et al., more than half of the nurses working in both wards admitted that they were not provided with adequate personal protective equipment to combat COVID-19.

Our study was not the only one to look at burnout among Polish healthcare workers. Tomaszewska et al. [29] aimed to assess the level of stress and burnout among nurses working with patients with COVID-19. The study showed that almost 50% of the nurses interviewed felt stressed during every shift, more than 40%—sometimes. The biggest stressors were the enormous responsibility for the health and lives of patients, the overload of tasks, direct contact with COVID-19 patients and their families, and deteriorating relationships with colleagues.

In our study, we assessed not only nurses but also doctors and paramedics. Rozhdestvenskiy et al. [31] surveyed a group of Russian doctors online. Like us, they used the MBI questionnaire, but other version (MBI-Human Services Survey for Medical Personnel, HSS-MP). The respondents were divided into two groups—the first group consisted of doctors working with patients with COVID-19, while the second group consisted of doctors who had no contact with such patients. Analysis of the results showed that emotional exhaustion and depersonalisation were higher among doctors who worked with COVID-19 patients. Similar results were obtained by Orrù et al. [32], who conducted the study among health professionals from 45 countries in 5 continents. The authors also pointed out that the phenomenon of burnout can contribute to the occurrence of unprofessional behaviour, which significantly reduces the quality of healthcare and thus affects the decline in patient satisfaction. This is why it is so important to ensure the mental well-being of doctors working in the profession.

Our research showed that female healthcare workers appeared to be at higher risk of job burnout than male healthcare workers, suggesting possible gender differences in work experiences or ways of coping with stress. Similar to our study, other researchers also found that the female gender was more susceptible to burnout, especially during the COVID-19 pandemic [34, 35].

It should also be emphasised that in our study, the risk of burnout was higher among nurses than among doctors and paramedics, which is also shown by other studies [27, 35]. All this (gender, profession, and working time) should be taken into account in interventions to prevent burnout.

### 4.1. Implications for Policy and Research

These findings highlight the need for further research on burnout among health professionals and the need for effective interventions to promote well-being and prevent burnout in this group. In addition, there is a need to support those who are already experiencing burnout to move towards a state of health and life satisfaction.

Counteracting burnout in the health service should be one of the main goals of public health policy. The pandemic situation is associated with an increased risk of burnout among doctors, nurses, and paramedics. Therefore, the experience gained during the last pandemic and the results of the research conducted should influence the preparation and development of appropriate procedures in the event of future pandemics.

It should also be remembered that nurses are the professional group most exposed to burnout. It is also associated with the lowest levels of life satisfaction. Therefore, activities to prevent burnout should pay particular attention to this group of health workers.

### 4.2. Strengths and Limitations of the Study

The main strength of the study was the large examined group. An additional value was the longitudinal analysis carried out in three different years, which made it possible to observe changes over time and to consider possible trends in life satisfaction and occupational burnout.

However, the study had some limitations. There may be other important factors affecting job burnout and life satisfaction, such as levels of social support, that were not included in the analysis. The SWLS and MBI scales are based on self-reports, which could be subjective. It is also worth noting the cohort effect: although the survey was longitudinal, it did not follow the same people over time, so the observed changes could be due to differences between cohorts and actual changes over time. Finally, the results may not be representative of health professionals outside the Silesian region or in other cultures.

It is worth noting that some common biases (such as social desirability bias) were limited by the online survey, which ensured anonymity of respondents.

## Figures and Tables

**Table 1:** Results of the Satisfaction with Life Scale (*X* ± SD, points).

	2020	2021	2022	*p*
Doctors	23 ± 1.5	21 ± 1.4	20 ± 1.6	0.001
Nurses	19 ± 1.7	17 ± 1.8	18 ± 1.9	0.01
Paramedics	22 ± 1.6	20 ± 1.7	21 ± 1.8	0.01

**Table 2:** Results of the Maslach Burnout Inventory scale (*X* ± SD, points) presented as total score (sum of points for each item in the given subscale) and average score (sum of points divided by number of items for each subscale).

Calculations	Medical profession	2020	2021	2022	*p*
*Emotional exhaustion (EE)*					
Total score	Doctors	21.0 ± 6.5	22.5 ± 7.0	21.5 ± 6.0	0.001
	Nurses	24.0 ± 6.0	25.5 ± 4.5	24.5 ± 5.5	0.001
	Paramedics	20.0 ± 5.5	21.0 ± 5.0	20.5 ± 6.0	0.001
Average score	Doctors	4.2 ± 1.3	4.5 ± 1.4	4.3 ± 1.2	0.001
	Nurses	4.8 ± 1.2	5.1 ± 0.9	4.9 ± 1.1	0.001
	Paramedics	4.0 ± 1.1	4.2 ± 1.0	4.1 ± 1.2	0.001

*Depersonalization (DEP)*					
Total score	Doctors	17.5 ± 5.0	18.5 ± 5.5	18.0 ± 5.0	0.001
	Nurses	20.0 ± 5.5	21.5 ± 6.0	20.5 ± 5.0	0.001
	Paramedics	16.5 ± 5.0	17.5 ± 5.5	17.0 ± 5.0	0.001
Average score	Doctors	3.5 ± 1.0	3.7 ± 1.1	3.6 ± 1.0	0.001
	Nurses	4.0 ± 1.1	4.3 ± 1.2	4.1 ± 1.0	0.001
	Paramedics	3.3 ± 1.0	3.5 ± 1.1	3.4 ± 1.0	0.001

*Sense of reduced professional accomplishment (SRPA)*					
Total score	Doctors	16.8 ± 7.2	16.2 ± 6.6	15.6 ± 6.0	0.01
	Nurses	14.4 ± 6.0	19.2 ± 6.6	18.6 ± 6.0	0.01
	Paramedics	15.0 ± 6.0	14.4 ± 5.4	13.8 ± 4.8	0.01
Average score	Doctors	2.8 ± 1.2	2.7 ± 1.1	2.6 ± 1.0	0.01
	Nurses	3.0 ± 1.0	3.2 ± 1.1	3.1 ± 1.0	0.01
	Paramedics	2.5 ± 1.0	2.4 ± 0.9	2.3 ± 0.8	0.01

## Data Availability

Data will be made available on request to the authors.
