# Correction to “Burnout and Life Satisfaction among Healthcare Workers Related to the COVID-19 Pandemic (Silesia, Poland)”

**DOI:** 10.1155/bn/9869136

**Published:** 2025-09-29

D. Łaskawiec-Żuławińska, M. Grajek, K. Krupa-Kotara, et al., “Burnout and Life Satisfaction among Healthcare Workers Related to the COVID-19 Pandemic (Silesia, Poland),” *Behavioural Neurology* 2024 (2024): 9945392, https://doi.org/10.1155/2024/9945392.

In the article titled “Burnout and Life Satisfaction among Healthcare Workers Related to the COVID-19 Pandemic (Silesia, Poland),” author Hasan Karacan was affiliated to *Department of Eastern Languages and Literature, Cyprus Science University, Casaphani, Cyprus,* which is incorrect. The correct affiliation for this author is as follows:

Tourism Faculty, Cyprus Science University, Kyrenia, Cyprus

We apologize for this error.

